# Increased prevalence of some birth defects in Korea, 2009–2010

**DOI:** 10.1186/s12884-016-0841-z

**Published:** 2016-03-22

**Authors:** Dirga Kumar Lamichhane, Jong-Han Leem, Myungsook Park, Jung Ae Kim, Hwan Cheol Kim, Jin Hee Kim, Yun-Chul Hong

**Affiliations:** Department of Social and Preventive Medicine, School of Medicine, Inha University, Incheon, Korea; Department of Occupational and Environmental Medicine, School of Medicine, Inha University, 7-206 3rd St. Shinhung Dong, Jung Gu, Incheon, Korea; Department of Integrative Bioscience & Biotechnology, Sejong University, 209 Neungdong-ro, Gwangjin-gu, Seoul, 05006 Korea; Department of Preventive Medicine, College of Medicine, Seoul National University, Seoul, Korea

**Keywords:** Birth defects, Prevalence, Korea

## Abstract

**Background:**

Birth defects are a leading cause of neonatal and infant mortality, and several studies have indicated an increase in the prevalence of birth defects; more recent investigations have suggested that the trends of some defects are increasing in rapidly industrialized areas. This study estimates the prevalence rate and types of birth defects in Korea.

**Methods:**

This study used medical insurance benefit data of 403,250 infants aged less than one year from the National Health Insurance Corporation from seven metropolitan areas in Korea for 2009 and 2010.

**Results:**

The prevalence rate of birth defects was 548.3 per 10,000 births (95 % CI: 541.1–555.6), 306.8 among boys and 241.5 among girls. Anomalies of the circulatory system (particularly septal defects) were the most common (180.8 per 10,000), followed by defects of the genitourinary tract (130.1 per 10,000) (particularly obstructive genitourinary and undescended testis), musculoskeletal system (105.7 per 10,000), digestive system (24.7 per 10,000), and central nervous system (15.6 per 10,000).

**Conclusions:**

Relatively higher rates of some birth defects were found in the metropolitan areas. The high differences of birth prevalences for septal heart defects and undescended testis are probably due in part to progress in clinical management and more frequent prenatal diagnosis. Environmental exposure might play a critical role in the development of some birth defects. In attempting to describe the prevalence and spatio-temporal variations of birth defects in Korea, establishment of a registry system of birth defects and environmental surveillance are needed.

## Background

The prevalence of birth defects has continued to increase and has also led to a significant proportion of infant and childhood mortality, whereas the infectious causes are decreasing due to the extensive and successful use of prevention and control programs [1, 2]. Approximately 3 % of 134 million annual births worldwide are associated with a major structural disorder [3]. The prevalence of birth defects varies among countries and regions. For more than two decades in the United States, birth defects have been the major cause of infant mortality, affecting 3 % of live births and 2.55 % in Europe [4, 5]. The burden of birth defects in the South East Asian regions remains unknown due to a lack of national-level surveillance mechanism [2]. In Korea, the prevalence of birth defects was 2.86 % [6], and the increase in the prevalence of some defects is becoming a major public health concern.

It has been reported that the cause of 60 % of congenital birth defects is unknown and primary prevention is impossible, and approximately 20 % of congenital cases are caused by genetic disorders [7]. In addition, it has been suggested that multiple factors play an etiologic role in the development of birth defects; the potential risk factors include chemical pollutants, dietary imbalances, ionizing radiation, and infections [8]. Besides, the prevalence rate of birth defects has increasingly been used as an indicator of exposure to several kinds of teratogens, particularly pesticides and pharmaceutical substances [9].

In the last few decades, early research investigating the trends of birth defects has shown variation to the temporal changes in the prevalence of birth defects. The prevalence of several birth defects has increased over time, including, for example, heart defects, obstructive genitourinary defects, Down syndrome, and gastroschisis [10–12]. Neurological defects, particularly anencephaly and spina bifida, have shown a significant decrease over time in several areas due to supplementation of food with fortified folic acid, development of prenatal diagnosis and elective termination before 20 weeks of gestation [1, 2, 11, 13]. In addition, decreasing trends have also been reported for other birth defects such as club foot and cleft lip with or without cleft palate [11]. Research involving some birth defects, mainly undescended testis and hypospadias, in the petrochemical areas and non-industrialized areas has concluded that there is a significant difference in their prevalences based on environmental characteristics [14, 15]. Furthermore, it has been reported that resident area during pregnancy, threatened abortion history, medication history during the first trimester, alcohol consumption, mother’s age and weight during pregnancy and parental consanguinity are also related to birth defects [16–18].

Over the past 40 years, Korea has transformed itself into a vibrant capitalist economy with a rapid rate of urbanization, aggravating the environmental degradation. At the same time, it is likely that the pattern of birth defects is changing with time. Despite the fact that the overall prevalence of birth defects in Korea is lower or similar to that of most developed countries, the increase in the prevalence of some categories of birth defects still poses a major public health concern. Although several studies have examined the characteristics of birth defects, few studies have been conducted in Korea. The objectives of this study are to estimate the current prevalence and types of birth defects in Korea and to describe the prevalence of selected birth defects.

## Methods

The study protocol was approved by the institutional review board (IRB) of the University of Inha School of Medicine before the start of the study. We have participated in the health monitoring program for newborn babies supported by the Ministry of Environment, Korea. The Ministry of Environment asked the Korea National Health Insurance Corporation (NHIC) to allow us access to the insurance claims database. This study used the insurance claims database for 2009–2010 and estimated the prevalence of birth defects using data from seven metropolitan areas (Seoul, Pusan, Daegu, Incheon, Gwangju, Daejeon, and Ulsan). The need for consent was waived by the IRB, because we only use NHIC database without private individual information. NHIC collects information on the insurance society, type of medical care institution, episodes of hospital admission, birthday, day of first visit, days of visits, diseases code, total expenses, and other individual characteristics. Target population for analysis comprised 403,250 infants aged less than one year. Analysis of the medical insurance claims database of birth defects diagnosed during the first year after birth was performed to obtain information on the study subjects. Data on the same birth defect in the same patient from various organizations were assembled and reviewed. The possibility of multiple occurrences of birth defects in the same patient was considered. An infant or a fetus with more than one anomaly was counted once only based on the primary diagnosis. A total of 46,679 infants were selected for referring to birth defects code or subcode. When these results were further refined based on the reconfirmation of diagnoses, rule out diagnoses, disease name and exclusion of inappropriate cases, 35,697 study subjects from patients born from 2009 to 2010 were selected for final analysis. Birth defects were classified according to International Classification of Diseases, Tenth version (ICD-10). Cases of minor anomalies (Q32.0, Q67.0-Q67.8, Q68.0, Q68.3-Q68.5, Q76.0, Q76.5, Q82.5, Q83.3, Q84.5, Q95.0, Q95.1) treated as outpatients were excluded from further analysis. The birth defects code from Q00 to Q99 included 27,645 cases, and analysis of 69 major birth defects, which were used in some European Surveillance of Congenital Anomalies (EUROCAT) studies, reduced the number of subjects to 22, 111.

In addition, the prevalence of birth defects for 2009–2010 was compared with that of a similar study conducted 16 years previously [19]. In this study, 601,376 infants aged under one year were covered by medical insurance in 1993 and 601,459 in 1994. The ICD-9 codes were used to classify birth defects and codes 740–759.9 were investigated. Data from pharmacies, dental clinics, and oriental medical clinics were excluded due to unreliability of diagnosis and 44,305 study subjects were selected for final analysis. To identify the matching codes of birth defects between 1993–1994 and 2009–2010, ICD-10 was converted to ICD-9, using ICD10Data.com, and common defects were selected. According to this procedure, 26 different categories and subcategories of birth defects were selected.

Birth defect prevalence was calculated by dividing the numerator (registered cases of congenital anomalies) by the relevant denominator (the number of infants below one year of age among medical insurance dependents). The prevalence was expressed as the number of cases per 10,000 live births. The Poisson distribution was used to calculate 95 % confidence interval of birth defects prevalence. Statistical analyses were performed using Stata version 11.2, and alpha was set at *p* < 0.05.

## Results

### Birth defects in 2009–2010

The number of live births in the study areas was 403,250 from 2009–2010, with 196,532 in 2009 and 206,718 in 2010. Table 1 shows the prevalence rate of birth defects found in this study, reporting the prevalence rate of 548.3 per 10,000 births (95 % CI: 541.1–555.6). The gender-wise prevalences of birth defects according to the involved organ or system are shown in Table 2.

**Table 1:** Prevalence of birth defects in Korea, 2009–2010

Birth Defects (ICD-10)	Number of cases	Proportion (%) in birth defect	Prevalence per 10,000	95 % CI
Nervous system (Q00–07)	631	2.85	15.6	14.5 to 16.9
Anencephaly (Q00–002)	4	0.02	0.05	0.03 to 0.3
Spina bifida (Q05.0–05.9)	311	1.4	7.7	6.9 to 8.6
Encephalocele (Q01.0–01.9)	28	0.1	0.7	0.5 to 1.0
Microcephaly (Q02)	122	0.6	3.0	2.5 to 3.6
Holoprosencephaly (Q04.0–04.2)	53	0.2	1.3	1.0 to 1.7
Congenital hydrocephalus (Q03.0–03.9)	113	0.5	2.8	2.3 to 3.4
Eye, ear, face and neck (Q10–18)	241	1.1	6.0	5.2 to 6.8
Anophthalmos (Q11.0–11.1)	1	0.005	0.02	0.001 to 0.1
Microphthalmos (Q11.2)	18	0.1	0.4	0.3 to 0.7
Congenital cataract (Q12.0)	57	0.3	1.4	1.1 to 1.8
Absence of iris (Q13.1)	5	0.02	0.05	0.04 to 0.3
Congenital glaucoma (Q15.0)	29	0.1	0.7	0.5 to 1.0
Congenital absence of auricle (Q16.0)	16	0.07	0.4	0.2 to 0.6
Microtia (Q17.2)	115	0.5	2.9	2.4 to 3.4
Circulatory system (Q20–28)	9768	44.2	242.2	237.5 to 247.1
Common atrial trunk (Q20.0)	10	0.05	0.25	0.1 to 0.5
Translocation of great vessels (Q20.3)	72	0.3	1.8	1.4 to 2.2
Single ventricle (Q20.4)	45	0.2	1.1	0.8 to 1.5
Tetralogy of fallot (Q21.3)	167	0.8	4.1	3.5 to 4.8
Ventricular septal defect (Q21.0)	2536	11.5	62.9	60.5 to 65.4
Atrial septal defect (Q21.1)	4756	21.5	117.9	114.6 to 121.3
Pulmonary valve atresia/stenosis (Q22.0–22.1)	332	1.5	8.2	7.4 to 9.2
Tricuspid atresia/stenosis (Q22.4)	8	0.04	0.2	0.1 to 0.4
Ebstein’s anomaly (Q22.5)	25	0.1	0.6	0.4 to 0.9
Hypoplastic left heart syndrome (Q23.4)	10	0.05	0.25	0.1 to 0.5
Patent ductus arteriosus (Q25.0)c^a^	1617	7.3	40.1	38.2 to 42.1
Coarctation of aorta (Q25.1)	112	0.5	2.8	2.3 to 3.3
Aortic valve atresia/stenosis (Q23.0)	37	0.2	0.9	0.6 to 1.3
Total anomalous pulmonary venous connection (Q26.2)	41	0.2	1.0	0.7 to 1.4
Respiratory system (Q30–34)	750	3.4	18.6	17.3 to 20.0
Choanal atresia (Q30.0)	17	0.08	0.4	0.2 to 0.7
Cleft lip with or without cleft palate (Q36.0–37.9)	330	1.49	8.2	7.3 to 9.1
Cleft palate without cleft lip (Q35.1–35.9	403	1.8	10.0	9.0 to 11.0
Digestive system (Q38–45)	994	4.5	24.7	23.1 to 26.2
Oesophagus atresia with or without fistula (Q39–39.1)	66	0.3	1.6	1.3 to 2.1
Anorectal atresia/stenosis (Q42.0–42.3)	241	1.1	6.0	5.2 to 6.8
Small intestine atresia/stenosis (Q41.0–41.9)	142	0.6	3.5	3.0 to 4.2
Duodenal atresia /stenosis (Q41)	56	0.3	1.4	1.0 to 1.8
Other small intestine atresia/stenosis (Q41.1–41.9)	86	0.4	2.1	1.7 to 2.6
Hirschsprung’s disease (Q43.1)	311	1.4	7.7	6.9 to 8.6
Atresia of bile ducts (Q44.2)	86	0.4	2.1	1.7 to 2.6
Annular pancreas (Q45.1)	6	0.03	0.1	0.05 to 0.3
Genital organs (Q50–56)	1631	7.4	40.4	38.5 to 42.5
Undescended testis (Q53–53.9)^b^	1174	5.3	29.1	27.5 to 30.8
Hypospadias (Q54–54.9)	401	1.8	9.9	9.0 to 11.0
Epispadias (Q64.0)	0	0	0	0
Indeterminate sex (Q56–56.4)	56	0.3	1.4	1.0 to 1.8
Urinary system (Q60–64)	3619	16.4	89.7	86.8 to 92.7
Renal agenesis (Q60.0–60.6)	112	0.5	2.8	2.3 to 3.3
Extrophy of urinary bladder (Q64.1)	1	0.005	0.02	0.001 to 0.1
Renal dysplasid (Q61.4)	36	0.2	0.9	0.6 to 1.2
Cystic kidney (Q61.0–61.9)	280	1.3	6.9	6.2 to 7.8
Obstructive genitourinary defect (Q62.0–62.8, Q64.3)	1860	8.4	46.1	44.1 to 48.3
Congenital hydronephrosis (Q62.0)	1330	6.0	33.0	31.2 to 34.8
Musculoskeletal system (Q65–79)	4261	19.3	105.7	102.5 to 108.9
Reduction deformity, upper limbs (Q71.0–71.9)	20	0.09	0.5	0.3 to 0.8
Reduction deformity, lower limbs (Q72,0–72.9)	42	0.2	1.0	0.8 to 1.4
Total limb reduction defects (Q71.0–71.9, Q72.0–72.9, Q73.0–73.8)	66	0.3	1.6	1.2 to 2.1
Congenital hip dislocation (Q65.0–65.9)	2473	11.2	61.3	58.9 to 63.8
Club foot–talipes equinovarus (Q66.0)	87	0.4	2.2	1.7 to 2.7
Diaphragmatic hernia (Q79.0)	52	0.2	1.3	1.0 to 1.7
Polydactyly (Q69.0–69.9)	620	2.8	15.4	14.2 to 16.6
Syndactyly (Q70.0–70.9)	365	1.7	9.1	8.1 to 10.0
Arthrogrypposis multiplex congenital (Q74.3)	22	0.1	0.5	0.3 to 0.8
Craniosynostosis (Q75.0)	387	1.8	9.6	8.7 to 10.6
Jeunes syndrome (Q77.2)	0	0	0	0
Achondroplasia/Hypochondroplasia (Q77.4)	27	0.1	0.7	0.4 to 1.0
Omphalocele (Q79.2)	88	0.4	2.2	1.8 to 2.7
Gastroschisis (Q79.3)	12	0.05	0.30	0.2 to 0.5
Chromosomal abnormalities (Q90–99)	216	1.0	5.4	4.7 to 6.1
Trisomy 13 (Q91.4–91.7)	1	0.005	0.02	0.001 to 0.1
Trisomy 18 (Q91.0–91.3)	5	0.02	0.1	0.04 to 0.3
Down’s syndrome (Q90.0–90.9)	189	0.9	4.7	4.0 to 5.4
Turner’s syndrome (Q96.0–96.9)	7	0.03	0.2	0.1 to 0.4
Kleinfelter’s syndrome (Q98.0–98.4)	7	0.03	0.2	0.1 to 0.4
Wolff-Hirschron syndrome (Q93.3)	1	0.005	0.02	0.001 to 0.1
Cri-du-chat syndrome (Q93.4)	6	0.03	0.1	0.05 to 0.3
Total	22,111	100	548.3	541.1 to 555.6

^a^Birth weight of less than 2,500 g was excluded. ^b^Gestational age of less than 36 weeks was excluded

**Table 2:** Prevalence of birth defects in boys and girls according to involved organs or system

System	Number of cases		Prevalence per 10,000 and 95 % CI	
	Male	Female	Male	Female
Nervous system (Q00–07)	326	305	8.1 (7.2–9.0)	7.6 (6.7–8.5)
Eye, ear, face and neck (Q10–18)	140	101	3.5 (2.9–4.1)	2.5 (2.0–3.0)
Cardiovascular system (Q20–28)	4,761	5,007	118.1 (114.7–121.5)	124.2 (120.8–127.7)
Lip and palate (Q30–34)	373	377	9.3 (8.3–10.2)	9.3 (8.4–10.3)
Digestive system (Q38–45)	536	458	13.3 (12.2–14.5)	11.4 (10.3–12.4)
Urogenital system (Q50–64)	4,217	1,033	104.6 (101.4–107.8)	25.6 (24.1–27.2)
Musculoskeletal system (Q65–79)	1,903	2,358	47.2 (45.1–49.4)	58.5 (56.1–60.9)
Chromosomal anomalies (Q90–99)	115	101	2.9 (2.4–3.4)	2.0 (2.0–3.0)
Total	12,371	9,740	306.8 (301.4–312.2)	241.5 (236.8–246.4)

### Birth defects of the central nervous system

Four cases of anencephaly (0.05 per 10,000; 95 % CI: 0.03–0.3), 311 cases of spina bifida (7.7 per 10,000; 95 % CI: 6.9–8.6), 28 cases of encephalocele (0.7 per 10,000; 95 % CI: 0.5–1.0), 122 cases of microcephaly (3.0 per 10,000; 95 % CI: 2.5–3.6), 53 cases of holoprosencephaly (1.3 per 10,000; 95 % CI: 1.0–1.7), and 133 cases of congenital hydrocephalus (2.8 per 10,000; 95 % CI: 2.3–3.4) were registered with associated prevalence rates at birth of 8.1 per 10,000 births (95 % CI: 7.2–9.0) for males and 7.6 per 10,000 births (95 % CI: 6.7–8.5) for females.

### Birth defects of eye, ear, face and neck

It was found that 140 cases of the defects were recorded in boys (3.5 per 10,000; 95 % CI: 2.9–4.1) and 101 cases in girls (2.5 per 10,000; 95 % CI: 2.0–3.0). The most common were microtia (2.9 per 10,000; 95 % CI: 2.4–3.4), congenital cataract (1.4 per 10,000; 95 % CI: 1.1–1.8), and congenital glaucoma (0.7 per 10,000; 95 % CI: 0.5–1.0). One girl had anophthalmos, while five cases of absence of iris (two in males and three in females), 18 cases of microphthalmos (eight in males and 10 in females), and 16 cases of congenital absence of auricle (nine in males and seven in females) were reported.

### Birth defects of the cardiovascular system

Congenital anomalies of the circulatory system affected more infants born (44.2 % of all birth defects) in the study area than any other type of birth defects: 4,761 in boys (118.1 per 10,000; 95 % CI: 114.7–121.5) and 5,007 in girls (124.2 per 10,000; 95 % CI: 120.8–127.7). The three most common types of heart malformations were atrial septal defect (117.9 per 10,000; 95 % CI: 114.6–121.3), ventricular septal defect (62.9 per 10,000; 95 % CI: 60.5–65.4), and patent ductus arteriosus (40.1 per 10,000; 95 % CI: 38.2–42.1).

### Birth defects of lip and palate

The study found 403 cases of cleft palate without cleft lip (10 per 10,000; 95 % CI: 9.0–11.0), 164 in boys (4.1 per 10,000; 95 % CI: 3.5–4.7) and 239 in girls (5.9 per 10,000; 95 % CI: 5.2–6.7); 330 with cleft lip with or without cleft palate (8.2 per 10,000; 95 % CI: 7.3–9.1), 198 in boys (4.9 per 10,000; 95 % CI: 4.3–5.6) and 132 in girls (3.3 per 10,000;95 % CI: 2.7–3.9); and 17 with choanal atresia (0.4 per 10,000; 95 % CI: 0.2–0.7), 11 in boys (0.3 per 10,000; 95 % CI: 0.1–0.5) and six in girls (0.1 per 10,000; 95 % CI: 0.05–0.3).

### Birth defects of the digestive system

It was found that 536 cases of the defects were diagnosed in boys (13.3 per 10,000; 95 % CI: 12.2–14.5) and 458 in girls (11.4 per 10,000; 95 % CI: 10.3–12.4). Hirschsprung’s disease (7.7 per 10,000; 95 % CI: 6.9–8.6), anorectal atresia/stenosis (6.0 per 10,000; 95 % CI: 5.2–6.8), and small intestine atresia/stenosis (3.5 per 10,000; 95 % CI: 3.0–4.2) were mainly reported.

### Birth defects of the urogenital system

There were 5,250 reported cases of congenital anomalies of the urogenital system, resulting in the prevalence of 104.6 per 10,000 in boys (95 % CI: 101.4–107.8) and 25.6 in girls (95 % CI: 24.1–27.2). The most common anomalies were obstructive genitourinary defect (46.1 per 10,000; 95 % CI: 44.1–48.3), congenital hydronephrosis (33.0 per 10,000; 95 % CI: 31.2–34.8), undescended testis (29.1 per 10,000; 95 % CI: 27.5–30.8), and hypospadias (9.9 per 10,000; 95 % CI: 27.5–30.8).

### Birth defects of limbs and musculoskeletal system

Structural limb anomalies include dysplasia, reduction defects and duplication defects with supernumerary limb elements. Of the 4,261 cases (1,903 males and 2,358 females), congenital hip dislocation (61.3 per 10,000; 95 % CI: 58.9–63.8), polydactyly (15.4 per 10,000; 95 % CI: 14.2–16.6), craniosynostosis (9.6 per 10,000; 95 % CI: 8.7–10.6), and syndactyly (9.1 per 10,000; 95 % CI: 8.1–10.0) were most prevalent.

### Chromosomal anomalies

102 boys (2.5 per 10,000; 95 % CI: 2.1–3.1) and 87 girls (2.2 per 10,000; 95 % CI: 1.7–2.7) had Down syndrome, the most commonly reported congenital autosomal anomaly. Three boys and four girls had Turner syndrome, while seven boys had Kleinfelter syndrome.

### Prevalence of birth defects in 1993–1994 and 2009–2010

**Fig. 1:**
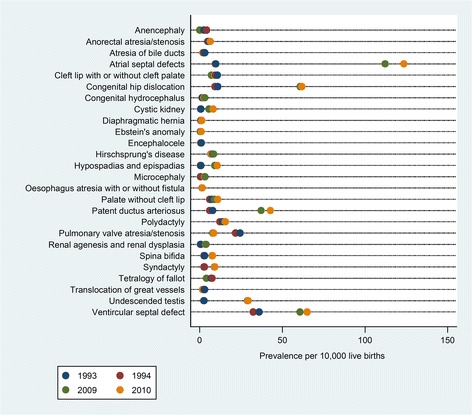
Prevalence for 26 selected birth defects for 1993, 1994, 2009 and 2010

In 1993–1994, the overall prevalence of birth defects per 10,000 infants less than one year was 368.3 (95 % CI: 364.9–371.8), and cardiovascular anomalies were the most common defects followed by musculoskeletal and gastrointestinal anomalies [19]. Table 3 shows the prevalence (95 % CI) for the selected birth defects based on the ranking of decreased prevalence according to percentage change in 2009–2010 with respect to 1993–1994, while Table 4 summarizes the prevalence based on the ranking of increased prevalence. It is seen that the prevalences of most defects, notably, hypospadias and epispadias, atrial septal defect, undescended testis, spina bifida and ventricular septal defect showed higher prevalences in 2009–2010. Figure 1 shows the pictorial variation to birth defect prevalences in 1993, 1994, 2009, and 2010. The prevalence estimates for anencephaly, anorectal atresia/stenosis, congenital hydrocephalus, cystic kidney, diaphragmatic hernia, encephalocele, Ebstein’s anomaly and renal agenesis and renal dysplasia tended to cluster together with occasional outliers. Atrial septal defect, patent ductus arteriosus, hypospadias and epispadias, undescended testis, ventricular septal defect and pulmonary valve atresia/stenosis exhibited considerable variation in prevalence across years, whereas spina bifida and syndactyly consistently appeared to have some of the least varying estimates.

**Table 3:** Prevalence of selected birth defects in 1993–1994 and 2009–2010, ranking of decreased prevalence

Order	Birth Defects	Prevalence per 10,000 live births (95 % CI)	
		1993–1994	2009–2010
1	Anencephaly	3.4 (3.1–3.8)	0.05 (0.03–0.3)
2	Pulmonary valve atresia/ stenosis	23.1 (22.2–24.0)	8.2 (7.4–9.2)
3	Tetralogy of fallot	7.3 (6.8–7.8)	4.1 (3.5–4.8)
4	Translocation of great vessels	2.9 (2.6–3.2)	1.8 (1.4–2.2)
5	Atresia of bile ducts	2.9 (2.6–3.2)	2.1 (1.7–2.6)
6	Encephalocele	0.9 (0.8–1.1)	0.7 (0.5–1.0)
7	Cleft lip with or without cleft palate	10.2 (9.6–10.8)	8.2 (7.3–9.1)

**Table 4:** Prevalence of selected birth defects in 1993–1994 and 2009–2010, ranking of increased prevalence

Order	Birth Defects	Prevalence per 10,000 live births (95 % CI)	
		1993–1994	2009–2010
1	Hypospadias and epispadias	0.7 (0.5–0.8)	9.9 (9.0–11.0)
2	Atrial septal defect	9.7 (9.1–10.3)	117.9 (114.6–121.3)
3	Undescended testis	2.6 (2.3–2.9)	29.1 (27.5–30.8)
4	Cystic kidney	0.7 (0.5–0.8)	6.9 (6.2–7.8)
5	Congenital hip dislocation	10.2 (9.6–10.8)	61.3 (58.9–63.8)
6	Microcephaly	0.5 (0.4–0.7)	3.0 (2.5–3.6)
7	Renal agenesis and renal dysplasia	0.6 (0.5–0.8)	3.7 (3.1–4.3)
8	Patent ductus arteriosus	7.0 (6.6–7.5)	40.1 (38.2–42.1)
9	Syndactyly	2.7 (2.4–3.0)	9.1 (8.1–10.0)
10	Spina bifida	2.8 (2.5–3.1)	7.7 (6.9–8.6)
11	Ventricular septal defect	34.1 (33.1–35.2)	62.9 (60.5–65.4)
12	Congenital hydrocephalus	1.6 (1.4–1.8)	2.8 (2.3–3.4)
13	Palate without cleft lip	6.8 (6.3–7.3)	10.0 (9.0–11.0)
14	Ebstein’s anomaly	0.5 (0.4–0.6)	0.6 (0.4–0.9)
15	Diaphragmatic hernia	1.0 (0.8–1.2)	1.3 (1.0–1.7)
16	Polydactyly	13.1 (12.5–13.8)	15.4 (14.2–16.6)
17	Anorectal atresia/stenosis	5.3 (4.9–5.7)	6.0 (5.2–6.8)
18	Hirschsprung’s disease	7.4 (6.9–7.9)	7.7 (6.9–8.6)
19	Oesophagus atresia with or without fistula	1.6 (1.4–1.8)	1.6 (1.3–2.1)

## Discussion

The overall prevalence of birth defects in our study area in 2009–2010 was 548.3 per 10,000, which is higher compared to the rates of Korea published three years previously [6]. In this study, cardiovascular system defects (242.2 per 10,000) were by far the most common defects, followed by defects of the urogenital system (130.1 per 10,000), musculoskeletal system (105.7 per 10,000), digestive system (24.7 per 10,000), and nervous system (15.6 per 10,000). Prevalence rates for these defects were grossly similar or slightly higher compared to the rates published previously [6, 14], except for atrial septal defects (117.9 per 10,000; 95 % CI: 114.6–121.3), ventricular septal defects (62.9 per 10,000; 95 % CI: 60.5–65.4), congenital hip dislocation (61.3 per 10,000; 95 % CI: 58.9–63.8), obstructive genitourinary defect (46.1 per 10,000; 95 % CI: 44.1–48.3), patent ductus arteriosus (40.1 per 10,000; 95 % CI: 38.2–42.1), undescended testis (29.1 per 10,000; 95 % CI: 27.5–30.8), congenital hydronephrosis (33.0 per 10,000; 95 % CI: 31.2–34.8), hypospadias (9.9 per 10,000; 95 % CI: 9.0–11.0), pulmonary valve atresia/stenosis (8.2 per 10,000; 95 % CI: 7.4–9.2), spina bifida (7.7 per 10,000; 95 % CI: 6.9–8.6), cystic kidney (6.9 per 10,000; 95 % CI: 6.2–7.8), and anorectal atresia/stenosis (6.0 per 10,000; 95 % CI: 5.2–6.8) for which the study found particularly high overall rates. Kim et al. [6] reported that the prevalence of birth defects was highest in the circulatory system followed by the musculoskeletal, digestive and urinary systems. Some studies reported the highest prevalence rate in the genitourinary system followed by the central nervous and digestive systems [20], while another study presented the order as the musculoskeletal, digestive, and genitourinary systems [21]. The variations to the reported frequencies could be due to the use of variety and subjectivity of classification criteria. In this study, the proportion of birth defects among males (56 %) is higher than in females (44 %). Similarly, Marden et al. [22] reported that the ratio of male to female infants was 58:42.

The elevated prevalence of heart defects including atrial septal defect and ventricular septal defect in the study is probably due to change in diagnostic method: routine use of echocardiography on newborns may have resulted in the identification of large numbers of defects. Progress in clinical management and more frequent prenatal diagnosis may have increased the prevalence of congenital heart defects, as also suggested by Khoshnood [23]. These results indicate the requirement of standardization of diagnostic and registration criteria for congenital heart anomalies. It is also suggested that environmental factors might play an important role in etiology of congenital heart defects. A meta-analysis study reported that the increased risk of atrial septal defect was related to exposure to PM_10_ (particulate matter 10) [24]. Likewise, some studies analyzing the association between traffic or the concentration of pollutants such as PM_10_ and carbon monoxide found that an increased risk of ventricular septal defect was due to maternal exposure to carbon monoxide and patent ductus arteriosus due to exposure to PM_10_ [25, 26].

The birth prevalence of undescended testis varies widely (1.4–31.7). The study found very high rates of undescended testis (29.1 per 10,000) in accordance with data from Canada (31.7 per 10,000) but in sharp contrast to the low rate of France (1.4 per 10,000) [27]. On the other hand, the birth prevalence for hypospadias (9.9 per 10,000) in this study was similar to that reported in France (9.8 per 10,000) [27].

In England, the prevalence of undescended testis increased by more than 60 % between the 1950s and 1980s, and the reported prevalence of hypospadias is 0.1–0.8 per 10,000 male births [28, 29]. A study conducted in North England found space-time clustering among cases of hypospadias, but not cryptorchidism [30]. The distribution of hypospadias cases may be predicted to exhibit spatial clustering if geographical varying environmental exposures are involved in their etiology. Space-time clustering occurs when an excess number of cases is observed within a small geographical area over a short period of time. In Spain, the frequency of hypospadias was 0.35 % and remained constant at this level for the past few decades, and a decreasing frequency was recorded only after 1996 [31]. The authors suggest that a radical change in the exposure that affected the whole country during that decade is a probable cause of that scenario in Spain. Unexpectedly, this sort of trend was not observed in the prevalence of any other birth defects. A study conducted in China reported an average annual increase in the overall prevalence of hypospadias of 7.34 % from 1996 to 2008, with geographical variation to increasing trends. The authors suggest that environmental exposure might play a critical role in the development of hypospadias [32]. Previous studies have suggested that the prevalence of undescended testis increased during the past half century in industrialized countries, and the spatial variation to the prevalence at birth has been indicated [30, 33]. Some studies have suggested that spatial and temporal trends of undescended testis and hypospadias might be associated with environmental factors, with minor involvement of genetic characteristics, and are among the most likely causes [33]. A number of animal-based studies have shown that perinatal exposure to exogenous oestrogens and anti-androgens may cause hypospadias, undescended testis, reduced sperm count and testicular cancers in males [33]. Chemicals such as pesticides, polychlorinated biphenyls, dioxins, naturally occurring plant estrogens, phthalates, bisphenol A and mycotoxins have been reported to affect reproduction in humans through endocrine-mediated processes [29]. However, a clear effect of an endocrine-induced disruption of chemical on reproductive organs in humans is yet to be established. Some studies found no strong epidemiological evidence to indicate a link between prenatal exposure to estrogen and malformation of male reproductive organs [28]. North and Golding found that a maternal vegetarian diet was a risk factor for hypospadias due to phytoestrogens in this type of diet [34]. Moreover, previous studies reported that the risk of birth defect varies according to the type of occupation [35–38]. A significant link was observed between maternal involvement in agricultural activities and an increased frequency of birth defects in the offspring. Pesticide exposure is likely in agricultural work even though direct handling of chemicals is not reported [36]. Therefore, the use of fertilizers, crop-preserving chemicals and use of spray in greenhouses can be major sources of exposure during agricultural activities. An increased risk among sons of mothers who were employed in gardening was also reported [38].

In particular, the prevalence of hypospadias and undescended testis in the study areas showed remarkably higher prevalence rates compared to the study conducted 16 years ago (Table 4). The tendency of increment was also higher than the national prevalence of undescended testis and hypospadias between 2000 and 2005. A study conducted in Korea reported an increased tendency from 5.01 to 17.43 per 10,000 persons for cryptorchidism and from 1.40 to 3.28 per 10,000 persons for hypospadias during that period [14]. Although the comparability of reported rates is poor, there may be an important underlying temporal variation which cannot be properly addressed until ascertainment and diagnostic criteria are standardized.

Hirschprung’s disease (7.7 per 10,000), the most common congenital gut motility disorder, is relatively easy to diagnose, thus, the relatively higher prevalence compared to North England (1.63 per 10,000) [39] is probably due to genuinely higher prevalence of this defect in the study areas. Although some studies have indicated that this disease is inherited, environmental factors may be responsible for sporadic cases [40].

With regard to nervous system defects, particularly spina bifida (7.7 per 10,000), the prevalence rate was comparable to that of Japan (6.2 per 10,000) but higher than found in the United States (3.8 per 10,000) [27]. The reason for this higher prevalence may be associated with dietary consumption of folate. It has been suggested that folate intake varies between populations, and lack of this nutrient is known to cause such defects [41]. A study conducted in Korea reported that fewer women (10.3 %) take folate in the periconceptional period [42].

The prevalence rate for Down syndrome (4.7 per 10,000) was particularly low in the study areas; however, other population-based studies had reported higher than this figure in Finland (31.0 per 10,000), France (27.3 per 10,000), and Japan (10.9 per 10,000) [27]. This syndrome can be diagnosed relatively easily prior to birth. Hence, it is likely that many fetuses with the syndrome might have aborted electively, and this may have contributed to the variable prevalence rates.

The existence of etiological heterogeneity for the birth defects has been described elsewhere in the literature [43]. A number of studies analyzed the association between the environmental and nutritional factors and the prevalence of undescended testis and spina bifida, respectively, and many studies found a significant association. In particular with undescended testis and hypospadias, evidence from experimental biological investigations and epidemiological studies have left little doubt that these defects can be a result of disruption of embryonic and gonadal development during fetal life. As the prevalence rates are higher in the study areas, the etiological impact of adverse environmental factors such as hormone disruptor, might be acting on susceptible genetic background, may be considered for further studies. However in order to obtain a dataset that is as complete as possible, systematic data gathering and surveillance of birth defects must be established in Korea. Registration of defects in elective terminations is very important as many defects are now identified prenatally and exclusion of aborted cases may complicate the identification of environmental factors [44]. Therefore, in attempting to describe the prevalence and spatio-temporal changes of birth defects, establishment of a registry system of birth defects, and environmental surveillance at national and local levels is needed for further study.

However, the results in this study must be interpreted in the context of some limitations. The study cannot avoid the methodological limitation of insurance based data analysis. Diagnostic criteria, coding and the timing for follow-up of outpatients might vary in the hospitals, and further validation of the diagnosis was difficult with the insurance database analysis due to lack of detailed records. For example, some newborns with birth defects might have died before entering the hospital, although the number of such patients should be rare. In addition, ICD-9 codes were used to specify birth defects for years 1993–1994, while ICD-10 was used to classify birth defects for 2009–2010. Two versions of disease classification differ substantially. The change to the tenth revision resulted in more detailed classification, with about 8,000 categories compared with about 5,000 categories in the ninth revision. The relatively large difference of some birth defects reported between 1993–1994 and 2009–2010 may be due to the switch from ICD-9 to ICD-10. This study used a comparable category of codes for 26 selected defects according to the ninth and tenth revisions. However, there may be an important underlying variation to case ascertainment and diagnostic criteria (inclusion or exclusion of minor cases) which may have given higher reported rates in some birth defects. A study found the comparability ratio of 0.9064 between ICD-10 and ICD-9 while explaining congenital anomalies and chromosomal abnormalities as a cause of death in the United States [45]. Nonetheless, this study is helpful in understanding increased prevalence of some important birth defects in Korea.

## Conclusion

Relatively higher rates of some birth defects were found in the metropolitan areas. The high difference of birth prevalences for septal heart defects and undescended testis are probably due in part to progress in clinical management and more frequent prenatal diagnosis. Environmental exposure might play a critical role in the development of some birth defects. In attempting to describe the prevalence and spatio-temporal variations of birth defects in Korea, establishment of a registry system of birth defects and environmental surveillance are needed.

## Abbreviations

CI: confidence interval
EUROCAT: European Surveillance of Congenital Anomalies
ICD-10: International Classification of Diseases, Tenth version
NHIC: National Health Insurance Corporation
